# Wuzang Wenyang Huayu decoction regulates differentially expressed transcripts in the rats' hippocampus after cerebral hypoperfusion

**DOI:** 10.1111/jcmm.14723

**Published:** 2019-11-09

**Authors:** Meng Xia, Ziyun Ruan, Ben Chen, Yunqiao Wang, Zengzi Zhou, Shiding Ren, Lin Wu, Nong Tang

**Affiliations:** ^1^ School of Basic Medicine Guangxi University of Chinese Medicine Nanning China; ^2^ Guangxi Key Laboratory for Foundational Research of Chinese Medicine Guangxi University of Chinese Medicine Nanning China; ^3^ The 3rd Xiangya Hospital Central South University Changsha China; ^4^ The First Affiliated Hospital to Guangxi University of Chinese Medicine Nanning China

**Keywords:** chronic cerebral hypoperfusion, Morris water maze test, transcriptomics, vascular dementia, Wuzang Wenyang Huayu decoction

## Abstract

The modified Wenyang Huayu decoction has been widely used to treat vascular dementia in China for thousands of years. We have previously proved that a modified version, Wuzang Wenyang Huayu decoction has the potential to be a more effective clinical treatment that can attenuate cerebral ischaemic injury. However, the global transcript profile and signalling conduction pathways regulated by this recipe remains unclear. This study established a two‐vessel occlusion rat model by bilateral common carotid artery occlusion. Two groups of rats were intragastrically treated Wuzang Wenyang Huayu 2.5 g/kg vs or Piracetam 0.15 g/kg for 2 weeks. Learning and memory abilities were measured with Morris water maze. Neuronal plasticity was observed by HE staining. Differentially expressed transcripts of rat hippocampus were analysed by transcriptomics with Illumina HiSeq2500 platform. Results showed that Wuzang Wenyang Huayu decoction significantly alleviated learning, memory deficits, coordination dysfunction and prevented hippocampus cellular injury; Results further revealed the increased gene expression in KEGG metabolic pathways (MT‐ND2. MT‐ND3, MT‐ND4, MT‐ND4L, MT‐ND5 and MT‐ATP8) and genes involved in signal transduction, carcinogenesis, immune system, endocrine system, nervous system etc (Results further revealed differential expression of genes involved in various systems, including MT‐ND2) Our discovery is likely to provide new insights to molecular mechanisms of Wuzang Wenyang Huayu regarding hippocampal transcripts in a murine vascular dementia model.

## 1 INTRODUCTION

Vascular diseases have been identified as a major cause of dementia. Thus, there is an urgent need to develop novel treatments for vascular diseases, as patient number is rapidly growing.^1^, ^2^ Previous evidence has shown that chronic cerebral hypoperfusion may cause cognitive impairment characterized by memory loss, cognitive deficits and vascular lesions in the brain.^3^, ^4^, ^5^ The consequence of brain blood hypoperfusion is accompanied with multiple pathophysiologies that therefore greatly complicates treatment. Diverse symptoms and complicated pathophysiology of brain blood hypoperfusion lead to great difficulty in developing treatments.

Vessel occlusion studies are frequently used to model cerebral hypoperfusion by creating ischaemic or oligemic injuries with various degrees of severity in murine brain. Two experimental approaches have evolved: (a) transient occlusion of the middle cerebral artery^6^ and (b) permanent occlusion of extracranial vessels, for example carotid arteries.^7^ To model chronic cerebral hypoperfusion, permanent bilateral occlusion of the common carotid arteries of rats is usually introduced. This procedure creates a model similar to human dementia developed because of vascular risk factors. As the vessel occlusion is permanent and long‐lasting, reperfusion injury does not occur. Therefore, it is easy to study under which the cerebral hypoperfusion is global, and thus, a distinct ischaemic core and penumbra region cannot be outlined, the damage to the nervous tissue is less dramatic.^8^

According to 《the Yellow Emperor's Internal Classic》^9^, ^10^, ^11^, ^12^: ‘The beginning of human beings, the first to become “Jin”, the Jin is the brain marrow’, the essence here refers to the ‘Shen’ essence. 《Medical Enlightenment》 also says that ‘the Shen is the pivotal for the wisdom’. The 《Jing Yue Quan Shu》^13^, ^14^ further emphasizes the important role of Shen essence by stating, ‘if the Shen is not enough then “yin” of the five internal organs cannot be nourished, and the “yang” of the five internal organs cannot be sent out’. Consequently, the ‘Jin’ from Shen is the essence and it is related to the five internal organs. In a word, traditional Chinese medicine believes that the essence of Shen deficiency is the fundamental cause of vascular dementia, thus the target to treat the dementia.

Based on evidence describe above, we designed and created the integrated prescription of Wuzang Wenyang Huayu decoction, which would supposedly enhance the ‘yang’ for the five internal organs to produce ‘Jin’. It has been used to treat patients with vascular dementia and shown to have significant curing effect.^15^, ^16^, ^17^ Based on our transcriptome results, genes that are being actively expressed at any given time which is particularly suitable for the understanding of molecular mechanisms of interactions between the dementia pathological change and the efficacy of the treatment. We, based on clinical application of Wuzang Wenyang Huayu decoction, attempted to understand the molecular mechanism underlined and were able to identify the transcripts expressed in dementia rats and treated rats. Among many more differential expressed genes, we prioritized several candidate genes that might play an important role in the regulation of preventive or reversing responses.

## 2 MATERIALS AND METHODS

### 2.1 Animals and VD model

250 g male Sprague Dawley rats purchased from Hunan Slack Jingda Experimental Animal Co. Ltd and housed in a well‐ventilated colony room having a 12‐hour (h) light/dark cycle with temperature of 22°C. All procedures were performed according to protocols approved by the Animal Care and Use Committee at Guangxi University of Chinese Medicine, in accordance with IASP Guidelines for the Use of Animals in Research.

To induce memory and spatial discrimination impairment through a ceased brain blood supply, bilateral carotid arteries occlusion (BCAO) was performed. Rats were anaesthetized with chloral hydrate (300 mg/kg, 10% chloral hydrate was reconstituted by using 0.9% physiological saline) by intraperitoneal injection to a deep anaesthesia (no reflex actions of hindlimb). A midventral cervical incision in the middle of the neck in the upper edge of the sternum (about 1‐1.5 cm long) was made. The submandibular gland was gently removed using the ophthalmic forceps, and the sternocleidomastoid muscle and sternohyoid muscle were pulled aside to expose the common carotid arteria. The bilateral common carotid arteries were doubly ligated with 4‐0 silk suture, the two sutures are next to each other and then the surgical wounds were sutured back with silk suture. The rats were returned back to its cage until fully recovered from anaesthesia. The antibiotics were administered for 3 days. Sham animals were subjected to all aspects of protocol (surgery, anaesthesia, recovery) except for the carotid artery occlusion.

### 2.2 Drug and treatment

The composition of the decoction has been seen in our previous report^18^; in brief, the component of the decoction Wuzang Wenyang Huayu contains (gram): rhizoma typhonii (fuzi, 15), rhizoma zingiberis (ganjiang, 15), radix morindae officinalis (bajitian, 15), ramulus cinnamomi (guizhi, 15), rhizoma pinelliae preparatum (fabanxia, 15), rhizoma acori tatarinowii (shichangpu 15), radix notoginseng (sanqi, 15), herba epimedii (yinyanghuo, 15), radix ginseng (raw ginseng, 15) and radix et rhizome rhei (dahuang, 6), supplied from the First Affiliated Hospital of Guangxi University of Chinese Medicine (authenticated by the edition of the Chinese Pharmacopoeia 2010). The further preparation of the procedures mainly includes: diluting and refluxing two times, that is adding 10 times of water and refluxing for 2 hours in the first time, then adding eight times of water and refluxing for 1.5 hours, followed with filtering the liquid and concentrating to a raw herbs concentration of 1 g/mL and storing it at 4°C. The reference drug was used with piracetam (0.8 g, Northeast Pharmaceutical Group Shenyang First Pharmaceutical Co., Ltd., batch number: National Pharmaceutical Standard H20030997) and diluted with saline to 0.1 g/mL when used.

Of total 47 rats used in the study, 10 rats were randomly assigned into sham, and 37 rats were received BCAO in which bilateral common carotid arteries (BCCA) were occluded by ligation with 4‐0 silk sutures. There were total 10 rats dead during/after procedures, the rest of survivals were randomly assigned to nine rats for each group, that is vascular dementia (VD), vascular dementia plus Wuzang Wenyang Huayu (VD‐WY), vascular dementia plus Piracetam (VD‐P). Rats after surgery were recovered for 2 weeks and then were administrated respectively with Wuzang Wenyang Huayu (2.5 g/kg) for VD‐WY, Piracetam (0.15 g/kg) for VD‐P, PBS for Sham, and VD.

### 2.3 Morris water maze trial and test

Morris Water Maze (MWM) test was performed in a circular water tank (120 cm in diameter) containing opaque water (22 ± 1°C) at a depth of 25 cm and dividing into four quadrants.^19^ A hidden escape platform (9 cm in diameter) was placed in the centre of one quadrant, with its surface 1 cm below the water. The rats were subjected to acquisition trial four times a day for five consecutive days from 23‐27 days after BCAO. During each trial, the rats were placed in water at four positions and the starting position was randomly selected. Each rat was allowed to swim for locating the hidden platform. Rats that failed to find the hidden platform within 60 seconds were placed on it for 30 seconds. The same platform location was used for all rats. On the sixth day, that is day 28, the platform was removed, and the rats were subjected to the spatial probe trial test for 60 seconds. The time and distance spent in the target quadrant were recorded. Each training ended when rat found the platform or by 1 minute. If the rat did not find the platform, they were guided to the platform by a stick and left on it for 1 minute. The swimming path and latency of every training was recorded. These measurements including swim distance, time spent and latency were re‐measured at day 36.

### 2.4 Tissue preparation

Rats were killed after the test with MWM. After being anaesthetized with chloral hydrate (360 mg/kg, intraperitoneal injection, 10% chloral hydrate was reconstituted by using 0.9% physiological saline), brains tissue including hippocampus from five rats in each group were removed and stored in liquid nitrogen for RNA extraction and sequencing screening. The tissue from rest rats were stored in 4% paraformaldehyde in 0.1 mol/L phosphate buffer overnight, which was followed by 30% sucrose solution in 0.1 mol/L phosphate buffer overnight. Brain sections (25 μm thick) were sliced for H‐E staining.

### 2.5 Transcriptomics test

#### 2.5.1 Construction of cDNA library and sequencing of transcriptome

The RNA of rat hippocampus was extracted by Trizol, the concentration and purity of the extracted RNA were detected by Nanodrop2000, the RNA integrity was detected by agarose gel electrophoresis and the RIN value was determined by Agilent 2100. After the sample met the requirements, the magnetic beads with poly‐T Oligo (dT) were used for A‐T base pairing with ploy A to the separate mRNA from total RNA for analysis of transcriptome information. The first cDNA chain was synthesized using a target fragment of mRNA as a template and randomly synthesized hexamers as primers. Then, buffer solution, dNTPs, RNase H and DNA polymerase I were added to synthesize the second cDNA chain. Next to the addition of the End‐Repair Mix, poly‐nucleotide tailing was used to add a poly(A) tail to the 3′ end of the cDNA to attach the Y‐shaped adaptor. The prepared DNA library was subjected to sequencing using an Illumina HiSeq2500.

#### 2.5.2 Original sequence quality control and assembly

After the sequencing completed, the raw data obtained by sequencing were first subjected for quality control analysis. A/T/G/C base content distribution statistics was examined whether or not presented with AT or GC separation, and base mass distribution statistics was examined the sequencing data quality and base error rate distribution.

The raw data were analysed by SeqPrep (https://github.com/jstjohn/SeqPrep) and Sickle (https://github.com/najoshi/sickle) for: (a) removal of the linker sequence (reads)in the sequencing, removing reads without inserts because of linker self‐ligation; (b) trimming off bases with low quality (mass value less than 20) at the end of the sequence (3′ end), if the remaining sequence still contained a mass value less than 10 the entire sequence was rejected, otherwise retained; (c) removing the reads containing N ratio of more than 10%; (d) discarding the adaptor and the sequence less than 20 bp after trimming. After those removed away the original linker sequence, low‐quality sequence and empty sequence, only high‐quality clean reads were obtained for subsequent data analysis.

#### 2.5.3 Analysis of transcriptome data

The filtered sequence was compared with the rat genome using software TopHat2 provided from http://tophat.cbcb.umd.edu . Performing the alignment, the distribution and coverage of the sequencing sequences on the reference genome and gene sequence were counted according to the comparison results. At the same time, the FPKM (Fragments per kilobase transcriptome per million mapped reads) calculated the expression level of the gene. The Blast2GO program was then used to compare the genes on the alignment with the GO (geneontology) database (http://www.geneontology.org/), and then the Goatools program was used to perform GO classification annotations on the major biological functions involved in these genes. Finally, the signal pathways or metabolic pathways of genes involvement were analysed with the KEGG (Kyotoencyclopedia of genes and genomes) database (http://wego.genomics.org.cn).

### 2.6 Differential expression and statistical analysis

According to the difference of gene expression levels among all groups, the differential genes were screened. The screening criteria for differentially expressed genes (DEG) were false discovery rate (FDR) <0.05 and log 2 |FC| ≥ 2, where FC (fold change) represents the ratio of expression between the two samples. Information annotation for differentially expressed genes, COG classification, GO functional classification and enrichment, and KEGG pathway enrichment analysis were aligned within databases. Data were expressed as the mean ± SEM and were analysed by student's *t* test or measurement ANOVA.

## 3 RESULTS

### 3.1 Behavioural tests

Morris water maze test, a currently and widely accepted tool for measuring the capability of learning, memorizing and spatial cognition,^15^ was performed to evaluate the impairment effect of BCAO and to evaluate the effect of the decoction of Wuzang Wenyang Huayu, which has been seen the clinical improvement in the patients who suffered with cognitive impairment and treated with the decoction at the traditional Chinese medical department.^17^, ^18^

**Figure 1:**
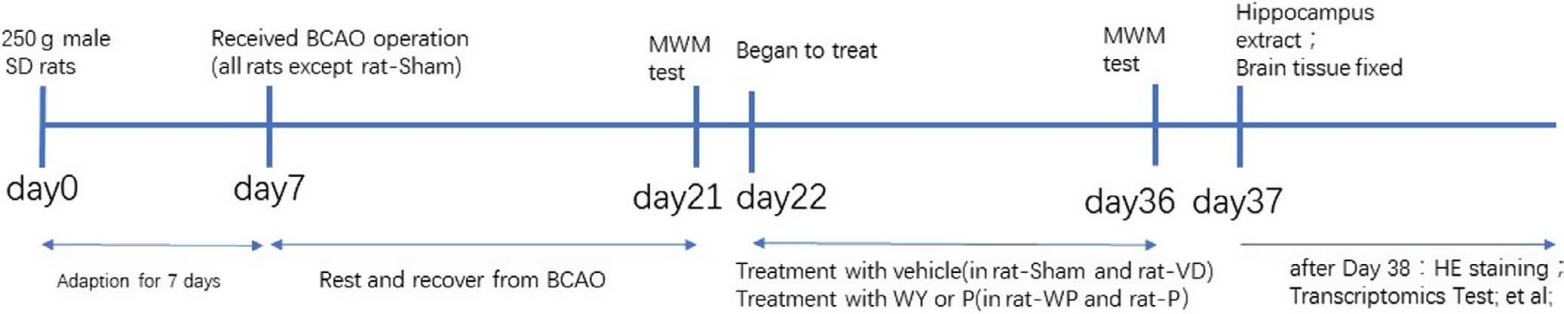
Workflow indication of procedures of modelling, treatment and measurements

**Figure 2:**
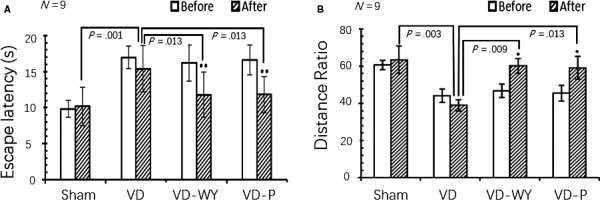
MWM trial and test in comparison with and without treatment after modelling. A, Escape latency calculated the time spent for rat locating the hidden platform within the time limit of 60 s, calculated as 60 s if not found out within 60 s. Before and after represent, respectively, for test at day 21 (before the treatment) and day 36 (after the treatment). The measurements of escape latency in “before” are as follows: 9.82 ± 1.16 (Sham), 16.96 ± 1.58 (VD), 16.19 ± 2.53 (VD‐WY) and 16.64 ± 2.07 (VD‐P). Note there is no statistically difference between pairs among all groups of VD vs VD‐WY, and vs VD‐P, and *P *> .05. The measurements of escape latency in “after” are as follows: 10.14 ± 2.65 (Sham), 15.38 ± 3.23 (VD), 11.78 ± 3.14 (VDWY) and 11.79 ± 2.52 (VD‐P). Note there is no statistically difference between VD‐WY and VD‐P. Among groups in comparing before and after, there is no statistical change observed in either Sham or VD. However, in VD‐WY, it was from 16.19 ± 2.53 in before and 11.78 ± 3.14 in after, decreased about 27%, **P = .005; in VD‐P it was 16.64 ± 2.07 in before and 11.79 ± 2.52 in after, decreased about 29%, **P < .001. B, Distance ratio calculated from the distance travelled in the target quadrant over the total path of the pool. Before and after represent respectively for the test at day 27 (before the treatment) and day 36 (after the treatment).The measurements of distance ratio in “before” are as follows: 60.67 ± 2.51 (Sham), 44.07 ± 3.58 (VD), 46.72 ± 3.68 (VD‐WY) and 45.50 ± 4.16 (VD‐P). Note there is no statistically difference between pairs among all groups of VD vs VD‐WY, and vs VD‐P, and *P* > .05. The measurements of distance ratio in “after” are as follows: 63.42 ± 7.30 (Sham), 39.03 ± 3.02 (VD), 60.09 ± 3.94 (VDWY) and 61.35 ± 6.19 (VD‐P). Note there is no statistically difference between VD‐WY and VD‐P. Among groups in comparing before and after, there is no statistical change observed in either Shamor VD. However, in VD‐WY, it was from 46.72 ± 3.68 in before and 60.09 ± 3.94 in after, increased about 22%, *P = .024; in VD‐P it was 45.50 ± 4.16 in before and 61.35 ± 6.19 in after, decreased about 259%, *P = .045. A comparison on trace tracking for different groups was also obtained as in supplement (Figure S1)

As seen from the work flow (Figure 1), all rats were experienced in learning swim at day 21 after 2 weeks' recovery from BCAO operation and one day before the treatment. All rats were trained in water for locating the platform in which the swim distance and locating for platform were recorded. As shown in Figure 2, the aggravated spatial learning deficit was clearly evident in all rats that were underwent BCAO. In versus to sham rats, the BCAO rats had an average of about 50% increasing in escape latency (n = 9, 9, 9, 9; *P* < .05) and an average of about 38% decreasing in distance ratio (n = 9, 9, 9, 9; *P* < .05), indicating the model establishment was satisfactory.

On the other hand, the VD rats which underwent BCAO without treatment behaved in significantly different to the rats which underwent treatment (VD‐WY and VD‐P). In escape latency, VD‐WY and VD‐P rats spent time were averaged about 12 seconds, shifting to that of sham rats (averagely about 10 seconds), whilst, VD rats spent time were averaged about 15 seconds (n = 9, 9, 9, 9; *P* < .05). The distance ratios were also improved in treatment rats which drastically increased for more than 35%; (n = 9, 9, 9, 9; *P* < .05). These results demonstrated that BCAO resulting in long‐term spatial learning‐memory impairment occurred in VD rats were partially reversed or improved by the treatments.

### 3.2 Injury assessment of the cerebral tissue

**Figure 3:**
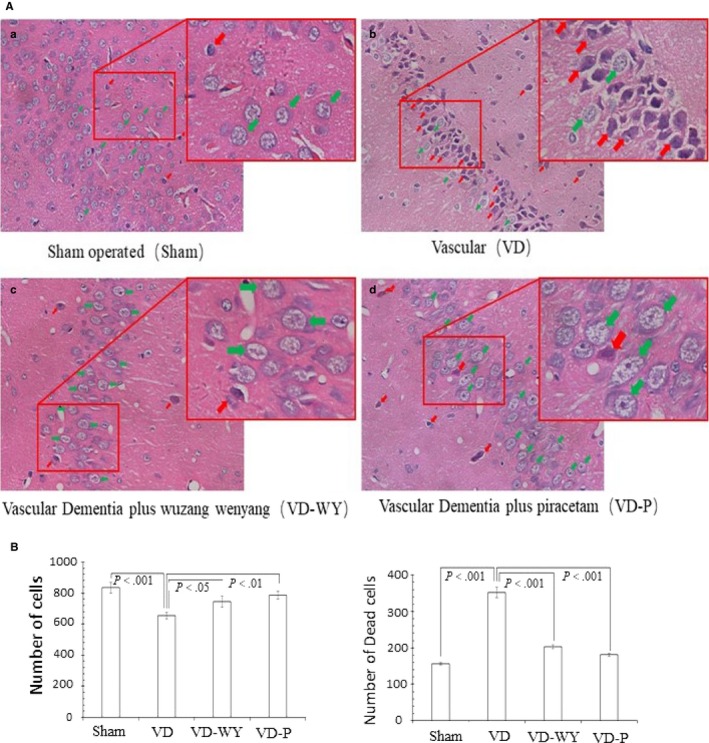
Hematoxylin and eosin staining hippocampus tissue A, H&E staining hippocampus tissue. a. Sham, b. VD, c. VD‐WY; d. VD‐P. In suite enlarges the corresponding region. Objective lens 20×. Scale bar: 100 μm. Note: Green arrows indicate normal status existed with tissue/cell contour integrated with nucleus and membrane; red arrows indicate dead or damaged with nucleus condense and aside. B, Statistical analysis of the images in figure A in which structure there were 250 regions of each group counted from each group

Brain tissue integrity and/or injury is a key factor relevant to the cognition, we examined the tissue using HE staining and calculated the tissue injury extent per brain sections as shown in Figure 3. The tissue injury was significantly increased in all three BCAO groups when compared with the sham group. Specifically, the severity of the BCAO damage was consistent with what seen from Morris water maze test, that is VD rats showed damage in severer, sham rats were in normal, and both VD‐WY and VD‐P rats presented an injury in moderate. Interestingly there were significant tissue damages happened in some VD‐WY rats but these individuals expressed cognition in a good function, suggesting functioning effect recovery is in advance of tissue injury recovery or there is a broader alternative circulation re‐established or alternatively compensated under the decoction of Wuzang Wenyang Huayu.

### 3.3 The sequencing of transcriptome quality control

To investigate the differential expression of transcriptomics upon the treatment, we chose five rats in each group for transcriptomics test. Each sample contained more than 6.3 GB clean date and a Q30 base percentage was greater than 90.7. The Clean Dates of each sample were sequence‐aligned with the designated reference genome, and the efficiency of the alignment was 95.51%‐96.78% as seen in Table 1.

**Table 1:** Statistics of transcriptomics sequencing data

Group	NO.	Clean reads	Clean bases	GC%	≥Q30%
sham	C1	42 214 392	6 296 532 145	49.36	90.99
	C2	55 936 552	8 352 951 973	49.76	91.89
	C3	47 618 166	7 099 571 367	49.45	90.7
	C4	47 626 166	7 114 667 735	49.89	91.79
	C5	55 809 138	8 334 224 103	50.06	91.85
VD	D1	47 439 398	7 078 785 421	49.33	91.81
	D2	60 472 116	8 985 010 124	46.55	91.63
	D3	50 467 226	7 509 707 442	48.72	91.06
	D4	50 475 832	7 505 286 996	45.92	91.37
	D5	49 531 418	7 393 037 858	45.78	91.45
VD‐P	P1	58 104 110	8 700 041 476	50.19	94.76
	P2	64 068 700	9 586 779 391	50.78	94.73
	P3	58 331 332	8 719 396 359	50.22	94.5
	P4	67 892 412	10 128 233 011	50.44	95.26
	P5	66 966 148	10 003 327 050	50.44	94.71
WD‐WY	WY1	56 381 088	8 422 048 496	49.28	91.99
	WY2	56 983 124	8 504 528 279	49.98	91.65
	WY3	62 908 760	9 418 212 635	48.93	94.81
	WY4	54 820 906	8 204 906 613	50.57	94.83
	WY5	60 845 360	9 102 803 325	49.64	94.41

Note

Clean reads means the total number of pair‐end reads in Clean Data; Clean bases means the total base pairs in Clean Data: the amount of CG is the GC content in Clean Data, namely the ratio of G and C base pairs in Clean Data; ≥Q30 means the ratio of the pair bases ≥30 in Clean Data.

### 3.4 Differentially expressed genes and the modulations by Wuzang Wenyang Huayu decoction

**Figure 4:**
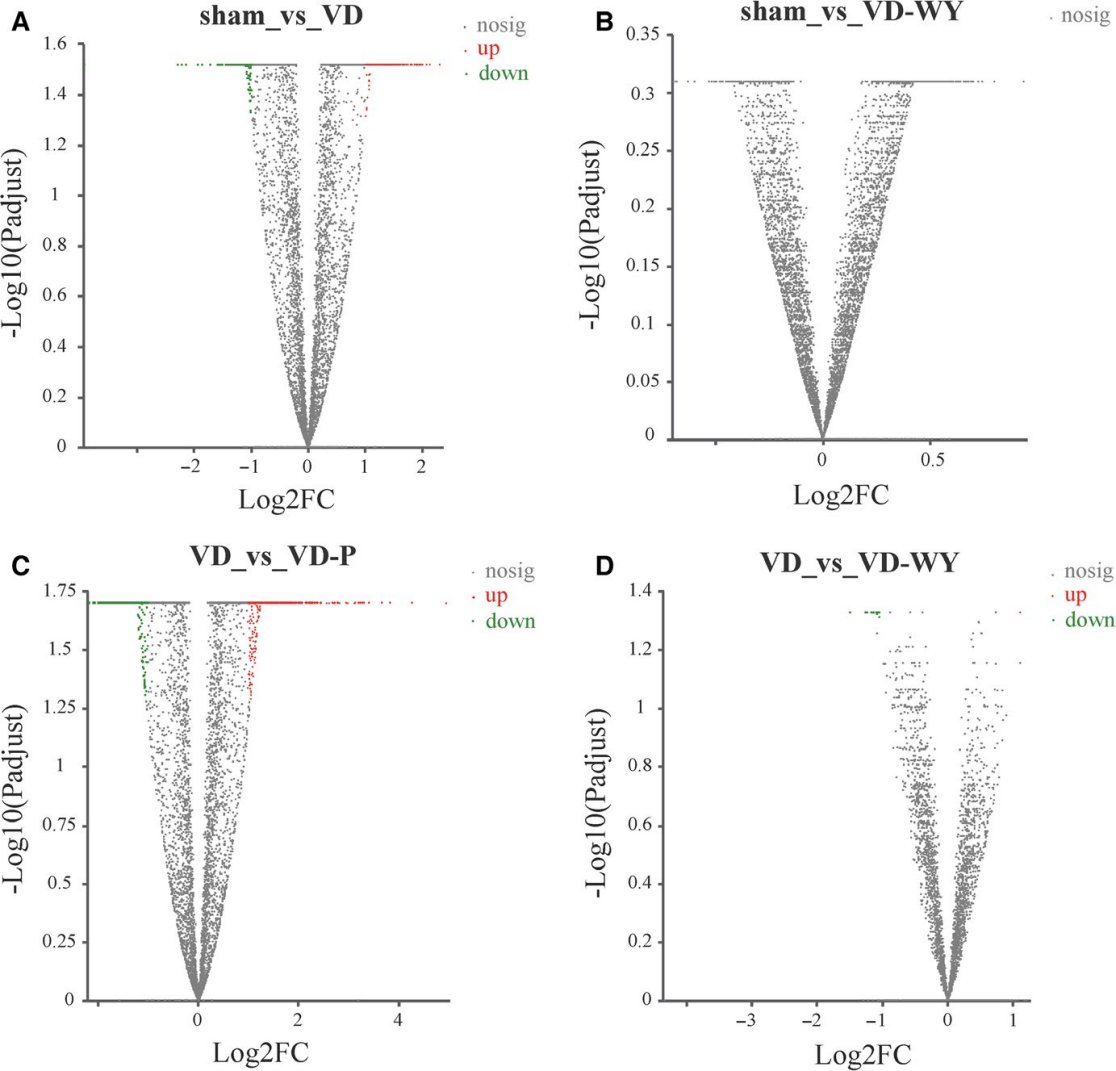
The distribution of differentially expressed genes in Sham group vs VD group (A), sham group vs VDWY group (B), VD group vs VD‐P group (C) and VD group vs VD‐WY group (D). The green and the red dots mean down‐regulated and up‐regulated differentially expressed genes, respectively

By using RSEM (http://www.biomedsearch.com/nih/RSEM-accurate-transcript-quantification-from/21816040.html), the transcripts from all groups including VD, sham, VD‐WY, and VD‐P were analysed by DEG. Compared with the sham, there were 511 up‐regulated genes and 424 down‐regulated genes in the VD rats, there were no significant differences in the VD‐WY rats (Figure 4A,B); compared with VD, there were 789 genes up‐regulated and 603 down‐regulated in VD‐P rats, and 1 gene up‐regulated and 15 genes down‐regulated in VD‐WY rats (Figure 4C,D).

### 3.5 Functional enrichment analysis of differentially expressed genes

**Figure 5:**
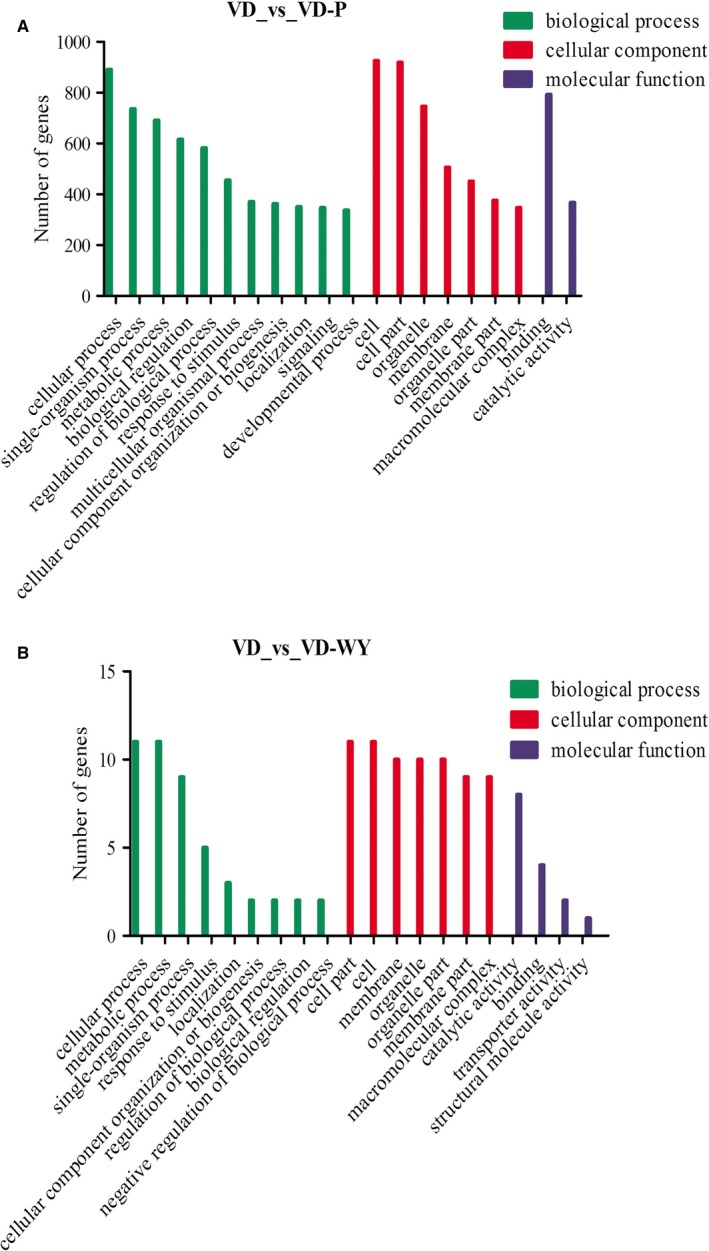
GO annotation classification statistics of differentially expressed genes in VD group vs VD‐P group (A) and VD group vs VD‐WY group (B). Results are summarized in three main GO categories: biological process (green), cellular component (red) and molecular function (blue)

Functional analysis of differentially expressed genes was first performed using GO analysis, as shown in Figure 5, those differentiated genes as mentioned in above were annotated to biological processes (BP), cellular components (CC) and molecular functions (MF), as well with the 20 subcategories.

However, there were great number of different between VD‐P vs VD and VD‐WY vs VD. GO analysis discerned those increased (1392) genes was mainly categorized in multicellular organismal process and signalling genes, attributed to biological process (Figure 5A). In molecular function analysis for understanding the differential gene of the VD, the difference between VD‐WY and VD‐P exists with 2 classes genes in transporter activity and structural molecule activity (Figure 5B).

### 3.6 KEGG annotations were further performed to compare VD respectively from VD‐WY rats and VD‐P rats

**Figure 6:**
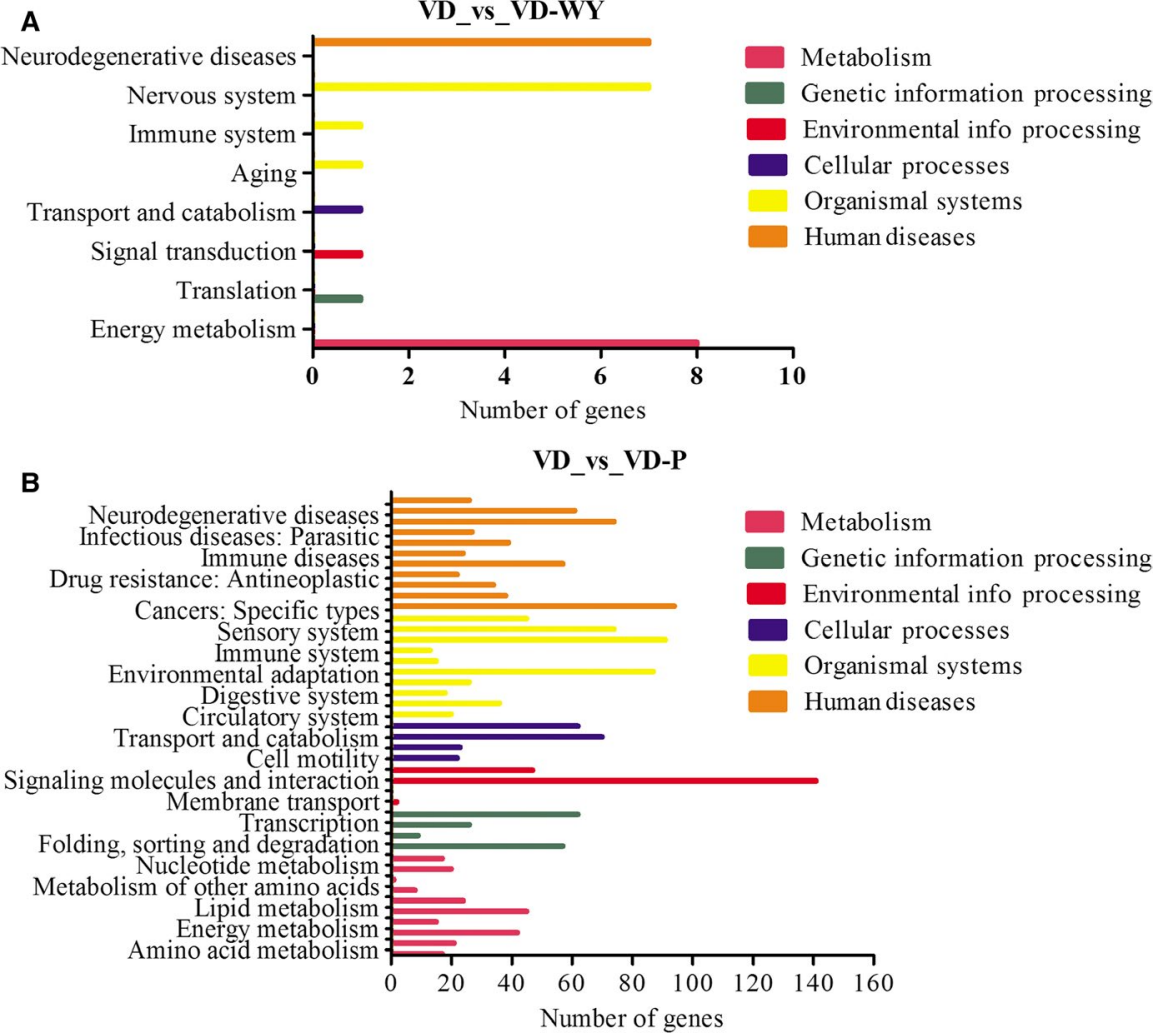
KEGG annotation classification statistics of differentially expressed genes in VD group vs VD‐WY group (A) and VD group vs VD‐P group (B). Results are summarized in six main KEGG categories: Metabolism (pink), genetic information processing (green), environmental information processing (red), cellular processes (blue), organismal systems (yellow), human diseases (orange)

As seen from Figure 6A, the gene differentially expressed in VD‐WY rats were mainly involved in KEGG metabolic pathways including energy metabolism (eight genes), nervous system (seven genes), neurodegeneration (seven genes), such as MT‐ND2. MT‐ND3, MT‐ND4, MT‐ND4L, MT‐ND5 and MT‐ATP8. As seen from Figure 6B, the gene differentially expressed in VD‐P rats was mainly involved the KEGG metabolic pathway including signal transduction (141 genes), carcinogenesis (94 genes), immune system (91 genes), endocrine system (87 genes), nervous system (74 genes), infectious or inflammation (74 genes) and cellular community—eukaryotes (70 genes).

## 4 DISCUSSIONS

In rat, bilateral carotid artery occlusion model has been well developed and established to mimic the effects of cerebral ischaemia that leads to vascular cognitive impairment dementia (VCID). In theory the bilateral carotid artery occlusion pathophysiology involves hypoxic hypoperfusion by a blocked vascular system^20^ resulting in injury to the white matter and hippocampal neuronal death leading to progressive decline in memory and cognitive function.^21^ This model has been used to test drugs to block the injury.^3^ Our data show that bilateral carotid artery stenosis (BCAS) with coils or ameroid constrictors produced slower development than BCAO, avoiding the acute ischaemia. But the fact is that use of reduction the diameter of the bilateral carotid artery for 2 months both functional and morphological criteria were not evident. The rat bilateral carotid artery occlusion has been shown a relative higher mortality compared with BCAS and also it has been noted that bilateral carotid artery occlusion is much more difficult to applied in mouse than in rat (data not shown). Therefore, the method of BCAO was chosen.

Test results from MWM showed that impaired learning and spatial memory from BCAO improved after the decoction to the same level as piracetam, supporting Wuzang Wenyang Huayu' effect of treating VD patients with deficit of learning memory.

Vascular dementia, including learning‐memory impairment, is one of the most severe symptoms of brain injury and has attracted attentions of numerous doctors and researchers.^22^, ^23^ Water maze is widely used to evaluate the learning‐memory function in many murine brain injury models.^24^, ^25^ Cognitive impairment after vascular dementia has been found the correspondingly pathological changes in brain, for examples increasing axon and myelin density in the corpus callosum and white matter bundles in the striatum, increasing oligodendrocyte and oligodendrocyte progenitor cell number in the corpus callosum, cortex and striatum and increasing synaptic protein expression in the cortex, striatum and hippocampus.^26^, ^27^ Our results show that the VD rats in MWM travelled less distance in the target quadrant and longer latency that indicates the modelling well established. More interestingly, using MWM to examine the effect of the decoction of Wuzang Wenyang Huayu, it has the similar effect like piracetam (commonly used in VD treatment in clinical) dramatically alleviated the learning and memory deficits. Based on the literatures and our experience, MWM measurement is a parameter for evaluating the learning‐memory function in studying traditional Chinese medicine including the complex of herbs, acupuncture and decoctions.

Cerebral ischaemia leads to delayed neuronal death in the hippocampal CA1 region.^28^ The cognitive deficits caused by ischaemia have been shown to be strongly correlated with neuronal plasticity in the hippocampus, and the pathological changes through several biologically plausible pathways in increasing axon, myelin density, white matter bundles, oligodendrocyte and oligodendrocyte progenitor cell number are mainly associated with RNA sequencing operated proteins.^29^ Thus, in this study, the global RNA transcripts profile of hippocampal cells was investigated using label‐free quantitative transcriptomics to explore the molecular events associated with cerebral hypoperfusion and the modulation of the concoction of Wuzang Wenyang. The result of transcriptomics analysis revealed 511 up‐regulated genes and 424 down‐regulated genes in the vascular dementia rats, obviously the dementia resulting in a broad impairment in brain presenting significant obstacle to treatment development. Interestingly, the concoction of Wuzang Wenyang in this experiment cannot only improve the function tested from MWM, but also partially reverse from the VD gene expression with 1 gene up‐regulated and 15 genes down‐regulated towards sham rats. In GO analysis, those differentiated genes are further annotated to 20 subcategories of biological processes, cellular components and molecular functions. Furthermore, this global transcripts study also finds out 2 classes of genes differentiated in transporter activity and structural molecule activity between VD‐WY and VD‐P. Based on KEGG pathway, gene with differential expression are involved in various pathways, including metabolism (eight genes), nervous system (seven genes) and neurodegeneration (seven genes) are expressed differentially, whereas, in VD‐P rats the genes differentially expressed in signal transduction (141 genes), carcinogenesis (94 genes), immune system (91 genes), endocrine system (87 genes), nervous system (74 genes), infectious or inflammation (74 genes) and cellular community—eukaryotes (70 genes). This is the first study to report the mechanism using transcriptomics based on traditional Chinese herbs.

Our previous investigation has found that Wuzang Wenyang might have therapeutic potential for the treatment of dementia caused by chronic cerebral hypoperfusion because of its protective effect on brain energy metabolic homeostasis and function. Combining previous and new finding we propose that the hypoperfusion protection of Wuzang Wenyang could be associated with the regulating differentially expressed transcripts in chronic cerebral hypoperfusion.

In summary, the present study indicated that Wuzang Wenyang decoction could protect against vascular dementia induced chronic cerebral injury in rats by modulating multiple transcripts. Result from transcriptome study, also suggested potential targets for the development of future treatments.

## CONFLICT OF INTEREST

The authors confirm that there are no conflicts of interest.

## AUTHOR CONTRIBUTIONS

Nong Tang and Lin Wu designed the study. Meng Xia, Ziyun Ruan and Ben Chen performed experiments. Yunqiao Wang, Shiding Ren analysed the data. Zengzi Zhou carried out critical revision of the manuscript for important intellectual content.

## ETHICAL APPROVAL

This study was approved and monitored by animal experiments ethical review committee of the Guangxi University of Chinese Medicine, Nanning, China.
