# Supplementary figures and images for: Wuzang Wenyang Huayu decoction regulates differentially expressed transcripts in the rats' hippocampus after cerebral hypoperfusion
Source: J Cell Mol Med. 2019 Nov 9;24(1):294–303. doi: 10.1111/jcmm.14723 (PMC6933406; doi:10.1111/jcmm.14723)

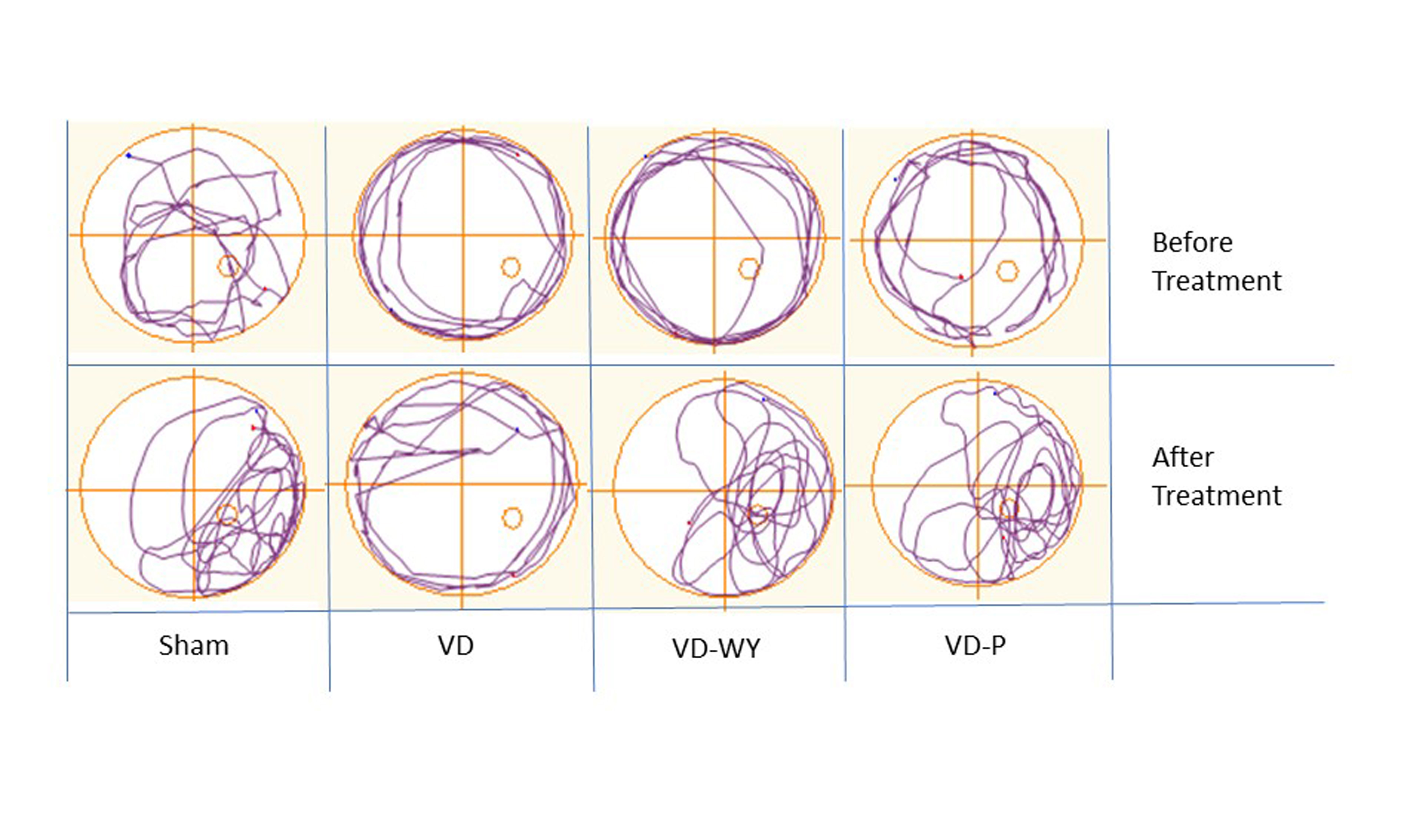

Supplement: Supplementary file 1 [file JCMM-24-294-s001.jpg]
